# Development and evaluation of a machine learning model for post-surgical acute kidney injury in active infective endocarditis

**DOI:** 10.3389/fcvm.2024.1425275

**Published:** 2024-12-05

**Authors:** XinPei Liu, SanXi Ai, RuiMing Yu, ChaoJi Zhang, Qi Miao

**Affiliations:** ^1^ Department of Cardiac Surgery, Peking Union Medical College Hospital, Beijing, China; ^2^ Department of Nephrology, Peking Union Medical College Hospital, Beijing, China; ^3^ Chief of Cardiac Surgery, Peking Union Medical College Hospital, Beijing, China

**Keywords:** active infective endocarditis, surgery, acute kidney injury, machine learning, clinical prediction model

## Abstract

**Introduction:**

Acute kidney injury (AKI) is notably prevalent after cardiac surgery for patients with active infective endocarditis. This study aims to create a machine learning model to predict AKI in this high-risk group, improving upon existing models by focusing specifically on endocarditis-related surgeries.

**Methods:**

We analyzed medical records from 527 patients who underwent cardiac surgery for active infective endocarditis from January 2012 to December 2023. Feature selection was performed using LASSO regression. These features informed the development of machine learning models, including logistic regression, linear and radial basis function support vector machines, XGBoost, decision trees, and random forests. The optimal model was selected based on ROC curve AUC. Model performance was assessed through discrimination, calibration, and clinical utility, with explanations provided by SHAP values.

**Results:**

Post-surgical AKI was observed in 261 patients (49.53%). LASSO regression identified 25 significant features for the models. Among the six algorithms tested, the radial basis function support vector machine (RBF-SVM) had the highest AUC at 0.771. The 15 most critical features were valve replacement, pre-operative hypertension, large vegetations, NYHA class, alcoholism, age, post-operative low cardiac output syndrome, TyG index, pre-operative creatinine clearance, cardiopulmonary bypass duration, intra-operative red blood cell transfusion, intra-operative urine output, pre-operative hemoglobin levels, and timing of surgery.

**Conclusion:**

Compared to standard cardiac surgery, AKI occurs more frequently and with a more complex etiology in surgeries for active infective endocarditis. Machine learning models enable early prediction of post-surgical AKI, facilitating targeted perioperative optimization and risk stratification in this distinct patient group.

## Graphical Abstract

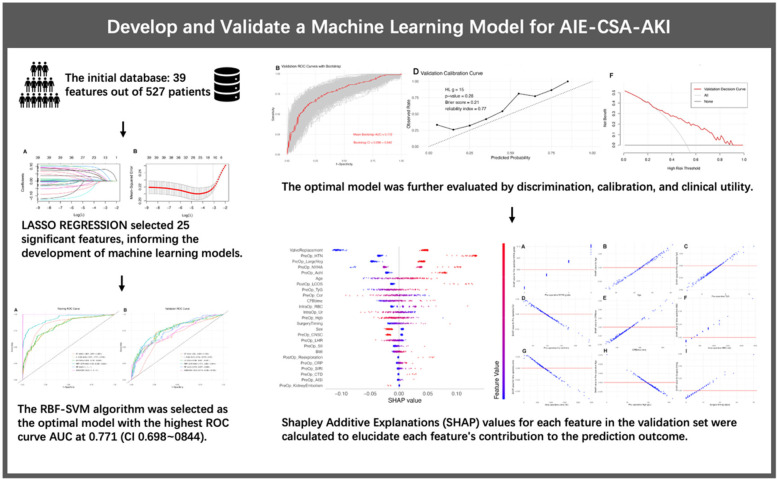

## Introduction

Acute kidney injury (AKI) is a common complication of cardiac surgery, with an incidence rate between 5% and 42%. Cardiac surgery-associated AKI (CSA-AKI) is linked to increased mortality and higher medical costs (1). Several models have been reported to predict the risk of CSA-AKI, including the Continuous Improvement in Cardiac Surgery Study score (2), the Cleveland Clinic Score (3), and the Mehta score (4). These models were developed from studies with sample sizes ranging from 30,000–50,000 patients and utilized the logistic regression algorithm to predict the risk of renal failure requiring dialysis after routine cardiac surgeries. In 2016, Birnie et al. developed a logistic regression model to predict AKI, as defined by KDIGO criteria, in 30,000 patients undergoing routine cardiac surgery, achieving an AUC of 0.74 (CI 0.72–0.76) (5). Recently in 2021, Jahan et al. generated machine learning models using four different algorithms to predict CSA-AKI and achieved AUCs outperforming the Cleveland Clinic score (6). However, these models, generated from data on patients undergoing CABG and/or valve surgeries, did not specifically consider patients with active infective endocarditis, which was not included as a feature in any previous models. Notably, the incidence of AKI in patients with active infective endocarditis (AIE-CSA-AKI) was reported to be as high as 59%–69%, approximately two to three times higher than that of CSA-AKI, and was associated with greater mortality rates, morbidity, and healthcare expenses (7, 8). The etiology of AIE-CSA-AKI is more complex, making it challenging to predict and resulting in worse outcomes. This underscores the necessity of developing and validating a predictive model specifically designed for AIE-CSA-AKI.

## Population and data processing

### Population

Between January 2012 and December 2023, a total of 544 patients underwent first-time cardiac surgery for active infective endocarditis at our institution, reoperation patients for prosthetic valve infective endocarditis (PVIE) were not included. Of these, 17 patients were excluded due to pre-operative renal failure requiring dialysis, leaving 527 patients eligible for inclusion in the study database. This single-center, retrospective, observational cohort study received approval from the Institutional Review Board of Peking Union Medical College Hospital (approval number: I-22PJ1016). All participants provided informed consent and signed the consent form.

### Data collection and preprocessing

Medical records were reviewed to collect 39 features for the database. These features included general information (sex, age, BMI, co-morbidities, alcohol and tobacco history), pre-operative assays (serum creatinine, hemoglobin, albumin, blood cell counts, fasting glucose, HDL, TG, CRP, PCT, and calculated indexes), pre-operative clinical characteristics (NYHA class, infective shock, vegetation size, peripheral embolisms, CNS complications), intra-operative data (left heart endocarditis, valve replacement, red blood cell transfusion, crystalloid infusion, urine volume, cardiopulmonary bypass duration), and major post-operative complications (LCOS, infective shock, re-exploration, atrial fibrillation). Detailed definitions, units, and data types for each feature are provided in Supplementary Table S1. For example, left heart endocarditis is defined as infective endocarditis affecting any left heart structure, large vegetation as any vegetation larger than 1 cm observed in pre-operative TTE, and post-operative LCOS as a cardiac index of less than 2.2 L/min/m^2^ measured by PiCCO. In addition, valve replacement refers to a situation where the defect in the valve tissue, after debridement, is so extensive that one or more valves are deemed unrepairable and must be replaced. This may also occur following an unsuccessful attempt at repair. The overall missing data rate among the 527 observations was 0.11%, with missing values imputed using the average or median values of the variables. Data processing, statistical analyses, and the development and validation of the machine learning model were conducted using R software (version 4.3.2).

### Definition of acute kidney injury

The development of postoperative AKI is defined according to the KDIGO criteria (9) within the first 7 days following surgery. It is characterized by an increase in serum creatinine by at least 50% within 7 days or an elevation of at least 0.3 mg/dl (equivalent to approximately 26.5 *µ*mol/L) within 48 h, compared to the baseline serum creatinine level measured preoperatively.

## Statistical analysis

### Feature selection

**Figure 1:**
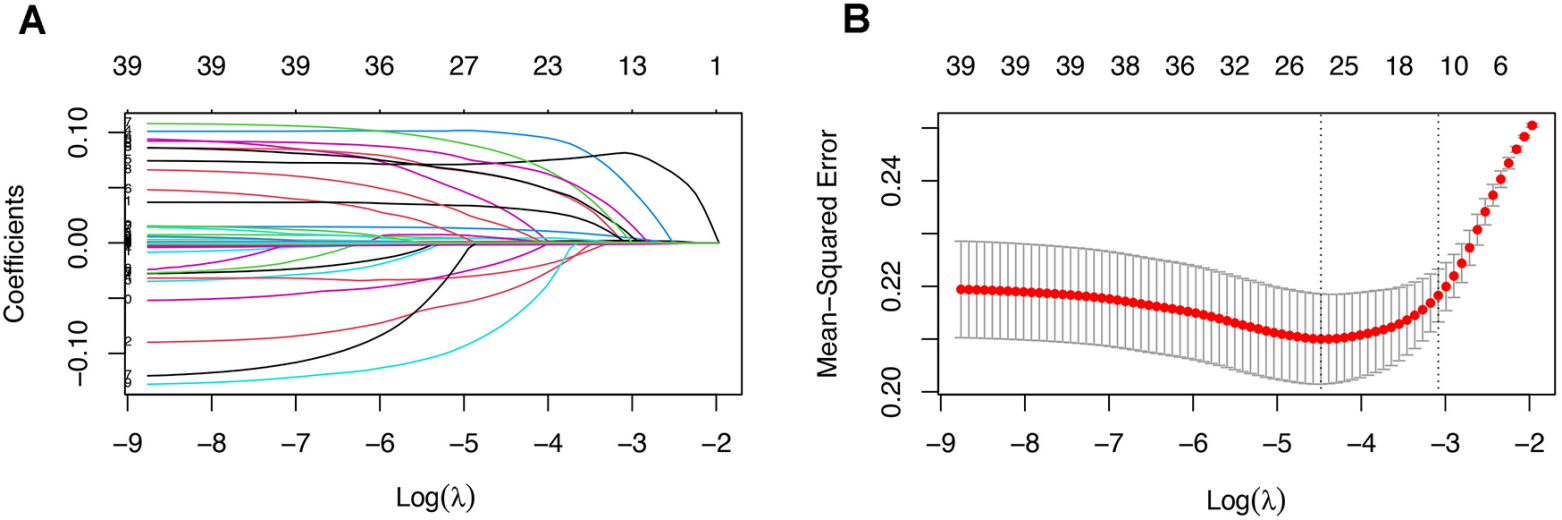
**(A)** Coefficient path of the LASSO regression. **(B)** Cross-validation plot of the LASSO regression, showing that 25 features were retained at the lambda value minimizing the mean squared error after 10-fold cross-validation.

A positive outcome was observed in 261 patients, suggesting that the number of features for model development should not exceed 26 to avoid overfitting. Consequently, we employed the Least Absolute Shrinkage and Selection Operator (LASSO) regression analysis for feature selection, as illustrated in Figure 1. After conducting 10-fold cross-validation, 25 features were retained at the lambda value that minimized the mean squared error. These features were deemed significant and utilized in the development of our machine learning models.

### Description and comparison

The dataset contained 527 observations, 25 features, and one outcome. Features were firstly compared between patients with or without CSA-AKI. Binary features were described in terms of percentages and assessed using the Chi-square test. Numeric features adhering to a normal distribution were summarized using means and standard deviations (SD) and evaluated with the independent *t*-test. Those not following a normal distribution were summarized using medians and interquartile ranges (IQR) and assessed with the Mann-Whitney *U* test. The dataset was then divided into a training set and a validation set in a 7:3 ratio. Descriptive statistics and intergroup comparisons were performed to confirm statistical consistency across the split datasets.

### Machine learning algorithms

We utilized different algorithms to develop predictive models based on the training set data. Discrimination was assessed in the validation set using the ROC curve. Six machine learning algorithms were evaluated: logistic regression (LR), linear support vector machine (L-SVM), radial basis function support vector machine (RBF-SVM), XGBoost, decision tree (DT), and random forest (RF).

The XGBoost model was trained using the “xgboost” R package, with the objective parameter set to “binary:logistic” and the evaluation metric for validation data as “auc”. The number of rounds was set to 100 with early stopping after 10 rounds. Default package settings were applied for other parameters. The DT model utilized the “rpart” R package, with the method set to classification and parameters at default. For the RF model, trained with the “randomForest” R package, “ntree” was set to 500. An automatic grid search determined the optimal parameters, with “mtry” set to 3 and “nodesize” to 1.

Given the sensitivity of SVM algorithms to parameter scaling, we performed maximum normalization on numeric and ordinal categorical features before training the SVM models. After normalization, the “e1071” R package was employed to train both the L-SVM and RBF-SVM models. For the RBF-SVM model, the “cost” parameter was set to 1 and “gamma” to 0.04. For the L-SVM model, “cost” was set to 0.01. After developing the models, we plotted the ROC curve for both training and validation sets of all six models and compared their AUCs. The model with the highest AUC and confidence interval (CI) was selected for further evaluation.

### Evaluation and interpretation of the best model

In addition to the ROC curve and AUC analysis, we employed 1,000 bootstrap samples to generate a bootstrap ROC curve, calculating the mean AUC and its CI. A calibration curve was plotted, and the Hosmer-Lemeshow test was performed to evaluate the model's calibration. The clinical utility was further assessed using decision curve analysis (DCA). Shapley Additive Explanations (SHAP) values for each feature in the validation set were calculated to elucidate each feature's contribution to the prediction outcome.

## Results

### Overall population

Medical records of 527 patients, who underwent cardiac surgery for active infective endocarditis at our institution, were reviewed. Among them, the median age was 47 years, and 68.5% were male. Hypertension was present in 23.7% of patients, while 23.1% had a history of alcoholism. Left heart infective endocarditis (IE) was diagnosed in 83.3% of cases, and 55.6% had large vegetations. The mean pre-operative TyG (triglyceride-glucose index) was 8.48. Mean creatinine clearance was 99.91 ml/min. The most common New York Heart Association (NYHA) class was class II (63.0%), followed by class III (21.8%). Valve replacement was performed in 62.8% of patients. The average cardiopulmonary bypass time was 139.5 min. Post-operatively, 8.0% of patients developed low cardiac output syndrome. AKI occurred in 261 patients (49.5%). Consequently, the number of features for model development should not exceed 26 to avoid overfitting.

### LASSO regression

As illustrated in Figure 1, we employed LASSO regression for feature selection. Out of 39 features included from the database, 25 were retained based on the lambda value that minimized the mean squared error, indicating their significance. All 25 features were depicted and compared between AIE patients with or without CSA-AKI (Table 1). These significant features were subsequently utilized in the development of our machine learning models, ensuring that the number of features was appropriate for the sample size. Consequently, these 25 significant features were used to train machine learning models. The coefficients of these significant features can be found in Supplementary Table S2.

**Table 1:** Variables in AIE patients with or without CSA-AKI.

Features	AKI group (261)	N-AKI group (266)	*P* Value
Age	52 [19]	42 [22]	0.067
BMI	22.59 (3.67)	22.00 (3.94)	0.074
CBPtime	152.26 (59.75)	127.06 (42.70)	<0.001
IntraOp_Crst	1,826.65 (593.37)	1,826.41 (475.32)	0.996
IntraOp_RBC	400 [600]	0 [400]	0.508
IntraOp_Ur	757.36 (441.24)	913.82 (428.54)	<0.001
LeftHeartIE	82.76%	83.83%	0.741
PostOp_Af	14.56%	9.77%	0.122
PostOp_IS	4.98%	0.75%	0.008
PostOp_LCOS	13.03%	3.00%	<0.001
PostOp_Reexploration	5.36%	0.75%	0.005
PostOp_dialysis	10.34%	0.75%	<0.001
PreOp_AISI	0.64 (1.24)	0.43 (0.56)	0.010
PreOp_Achl	28.35%	18.05%	0.005
PreOp_Alb	33.97 (6.27)	35.46 (5.90)	0.005
PreOp_CNSC	21.46%	24.44%	0.478
PreOp_CRP	46.13 (39.79)	37.57 (34.75)	0.009
PreOp_CTD	5.75%	2.26%	0.041
PreOp_Ccr	91.18 (39.81)	108.47 (33.20)	<0.001
PreOp_DM	11.11%	6.39%	0.056
PreOp_Embolism	37.55%	37.97%	0.921
PreOp_HTN	31.80%	15.79%	<0.001
PreOp_Hgb	99.97 (20.02)	109.09 (19.84)	<0.001
PreOp_IS	5.75%	0.75%	0.001
PreOp_KidneyEmbolism	1.92%	3.76%	0.312
PreOp_LHR	1.76 (1.14)	2.01 (1.21)	0.015
PreOp_LMR	3.47 (1.99)	4.08 (2.25)	0.001
PreOp_LargeVeg	61.30%	50%	0.009
PreOp_MHR	0.61 (0.48)	0.59 (0.45)	0.718
PreOp_NHR	8.70 (9.75)	8.06 (7.68)	0.398
PreOp_NLR	6.15 (6.54)	4.34 (3.53)	<0.001
PreOp_NYHA	2 [1]	2 [0]	0.168
PreOp_SCr	89.94 (55.09)	72.02 (33.17)	<0.001
PreOp_SII	1,229.61 (1,495.37)	968.70 (826.47)	0.014
PreOp_SIRI	3.21 (4.66)	2.03 (2.32)	<0.001
PreOp_Smk	30.65%	25.56%	0.195
PreOp_TyG	8.55 (0.45)	8,40 (0.48)	<0.001
Sex (Male)	72.41%	64.66%	0.055
SurgeryTiming	6 [5]	5 [4]	0.050
ValveReplacement	71.65%	54.14%	<0.001

Detailed definitions, units, and data types for each feature are provided in Supplementary Table S1.

### Development of the models

The dataset, comprising 527 observations, 25 features, and 1 outcome, was split into training and validation sets at a 7:3 ratio. The differences in these features between the two sets were tested (Table 2), and none were found to be significant (*P* > 0.05), indicating the consistency between the two sets.

**Table 2:** Consistency test between the training and validation sets.

Features	Train set (368)	Test set (159)	*P* value
AKI	48.37%	52.20%	0.48
Age	48 [24]	47 [21.5]	0.08
BMI	22.21 (3.78)	22.49 (3.90)	0.44
CPB time	140.95 (53.35)	136.27 (53.27)	0.36
IntraOp_Crst	1,838.06 (553.14)	1,799.84 (496.59)	0.43
IntraOp_RBC	400 [400]	0 [400]	0.78
IntraOp_Ur	858.54 (447.54)	784.91 (423.98)	0.07
LeftHeartIE	83.97%	81.76%	0.62
PostOp_Af	11.96%	12.58%	0.96
PostOp_IS	3.26%	1.89%	0.56
PostOp_LCOS	8.15%	7.55%	0.95
PostOp_Reexploration	2.45%	4.40%	0.35
PostOp_dialysis	6.52%	3.14%	0.18
PreOp_AISI	0.53 (0.80)	0.54 (1.27)	0.98
PreOp_Achl	23.10%	23.27%	1.00
PreOp_Alb	34.65 (5.88)	34.90 (6.68)	0.69
PreOp_CNSC	22.83%	23.27%	1.00
PreOp_CRP	40.74 (35.69)	44.30 (41.53)	0.35
PreOp_CTD	3.80%	4.40%	0.94
PreOp_Ccr	99.40 (36.24)	101.09 (40.66)	0.65
PreOp_DM	7.61%	11.23%	0.22
PreOp_Embolism	38.32%	36.48%	0.76
PreOp_HTN	22.28%	27.04%	0.29
PreOp_Hgb	104.95 (20.23)	103.70 (20.90)	0.53
PreOp_LargeVeg	56.25%	54.09%	0.72
PreOp_IS	2.99%	3.77%	0.84
PreOp_KidneyEmbolism	2.99%	2.52%	0.99
PreOp_LHR	1.91 (1.24)	1.82 (1.03)	0.46
PreOp_LMR	3.67 (1.86)	4.04 (2.67)	0.11
PreOp_MHR	0.61 (0.47)	0.58 (0.46)	0.61
PreOp_NHR	8.35 (8.71)	8.43 (8.91)	0.92
PreOp_NLR	5.15 (4.11)	5.44 (7.40)	0.64
PreOp_NYHA	2 [1]	2 [1]	0.29
PreOp_SCr	80.80 (45.72)	81.13 (47.46)	0.94
PreOp_SII	1,076.34 (949.80)	1,147.86 (1,668.26)	0.61
PreOp_SIRI	2.64 (3.47)	2.55 (4.26)	0.82
PreOp_Smk	29.08%	25.79%	0.51
PreOp_TyG	8.48 (0.47)	8.46 (0.49)	0.62
Sex(Male)	69.29%	66.67%	0.62
SurgeryTiming	6 [5]	6 [5]	0.45
ValveReplacement	63.86%	60.38%	0.51

Detailed definitions, units, and data types for each feature are provided in Supplementary Table S1.

**Figure 2:**
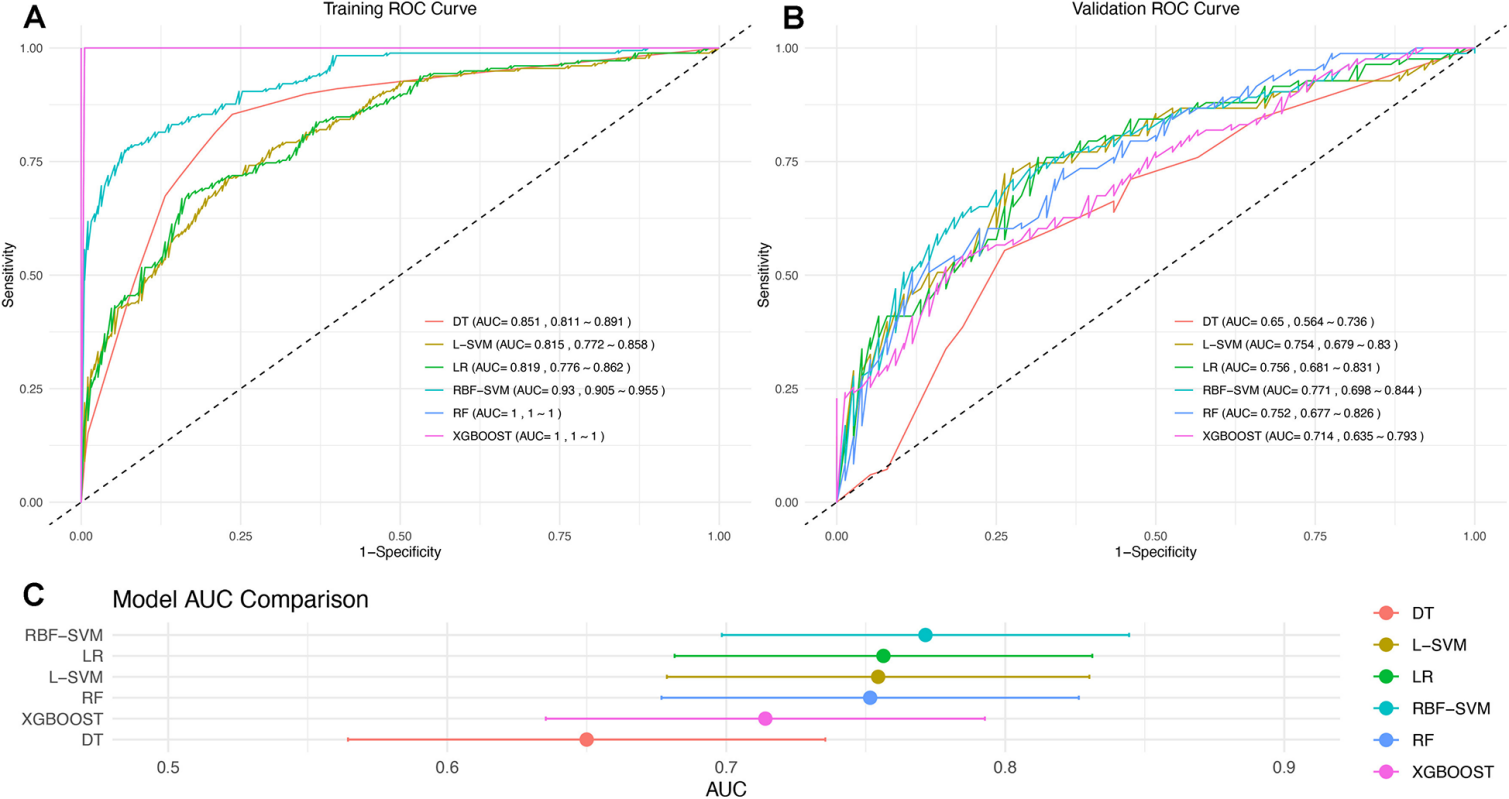
ROC curve for six machine learning algorithms developed from the **(A)** training and **(B)** validation sets. **(C)** Notably, the RBF-SVM model demonstrates superior performance, as indicated by its highest AUC value, and is emphasized for further evaluation and interpretation.

Six machine learning algorithms were employed to develop models using the training set. The sensitivity and specificity of these six models are listed in Supplementary Table S3. The ROC curves, AUC values, and CIs were compared, as shown in Figure 2. The RBF-SVM algorithm exhibited the highest AUC value and was, therefore, selected for further evaluation and interpretation. The.rds file of the specific RBF-SVM model has been uploaded as a supplementary file for further testing and application in the R environment. For all 25 features in the model, their definitions, units, and data types can be seen in Supplementary Table S1.

### Model evaluation

**Figure 3:**
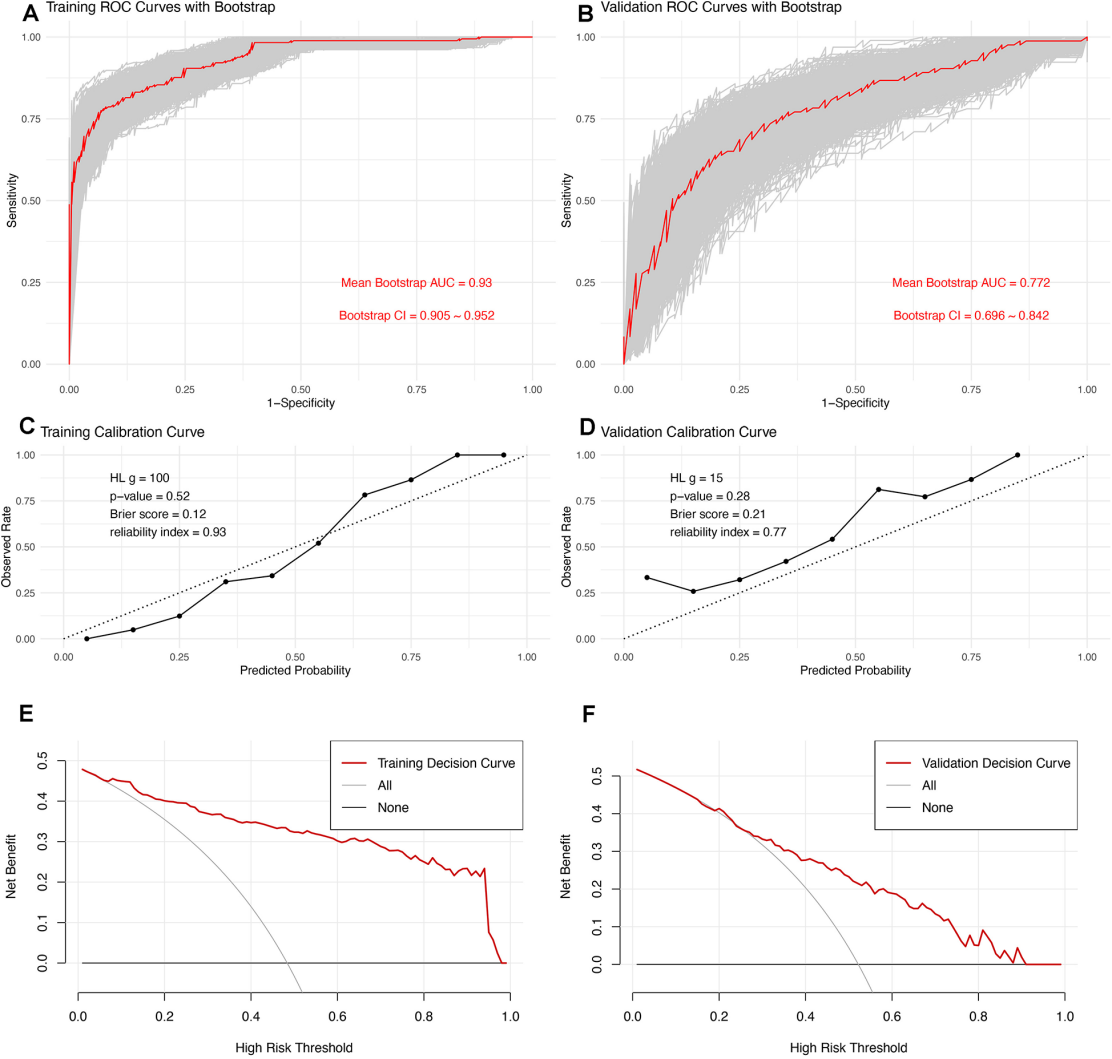
Comprehensive Evaluation of the Predictive Model: **(A,B)** ROC curves for the training and validation sets. For the validation set, the apparent AUC is 0.771 (CI, 0.698–0.844), and the mean bootstrap AUC is 0.772 (CI, 0.696–0.842), indicating excellent and robust model discrimination. **(C,D)** Calibration curves assess the model's accuracy in probability predictions across different thresholds. The curve for the validation set is slightly above the diagonal, suggesting marginally overestimated predictions. However, the model's calibration is deemed satisfactory, supported by a Hosmer-Lemeshow test *P*-value of 0.21, a Brier score of 0.21, and a reliability index of 0.77. (**E,F)** DCA evaluates the clinical practicability of the model, demonstrating that the model provides substantial clinical benefit across a range of decision thresholds.

To assess the model's discrimination, we employed 1,000 bootstrap samples to generate a bootstrap ROC curve, which was plotted alongside the apparent ROC curve (Figure 3A,B). The apparent AUC was 0.771 (CI, 0.698–0.844), while the mean bootstrap AUC was 0.772 (CI, 0.696–0.842), indicating that the model's discrimination is both good and robust. To evaluate the model's calibration, we used a calibration curve (Figure 3C,D). Notably, the curve for the validation set slightly exceeded the diagonal line, suggesting that the prediction probability was marginally higher in the validation set. However, the Hosmer-Lemeshow test yielded a *P* value of 0.21, the Brier score was 0.21, and the reliability index was 0.77, all of which suggest satisfactory model calibration. The Decision Curve Analysis (DCA) curve further indicated that the model possesses good clinical practicability (Figure 3E,F).

### Model interpretation

**Figure 4:**
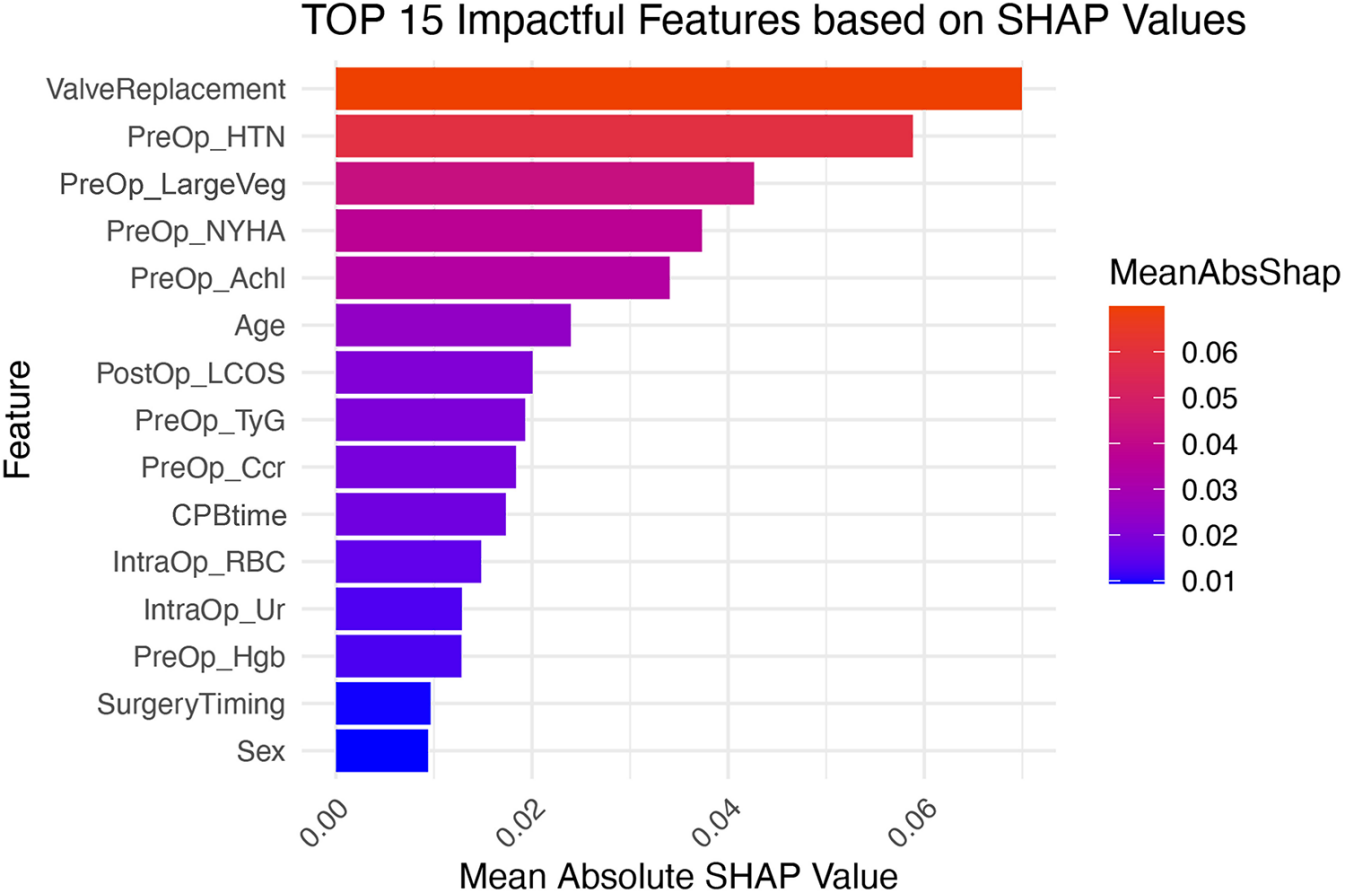
Bar chart displaying the top 15 important features influencing AIE-CSA-AKI, ranked by mean SHAP values. Features include valve replacement, pre-operative hypertension, large vegetations, NYHA class, alcoholism, age, post-operative LCOS, TyG, Ccr, CPB time, intra-operative red blood cell transfusion, intra-operative urine outputl, pre-operative hemoglobin, surgery timing, and sex.

**Figure 5:**
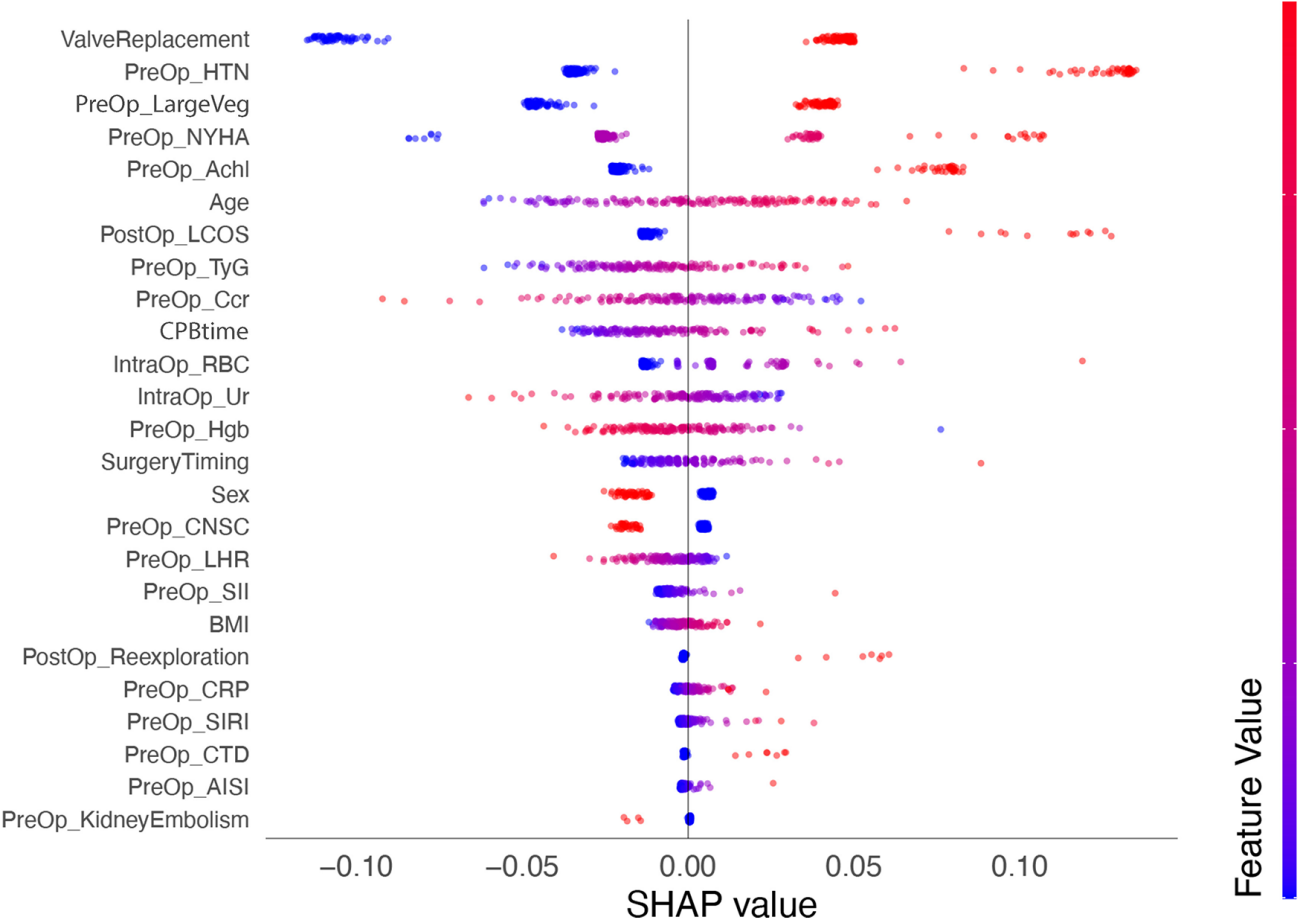
SHAP summary plot depicting the influence of various features on the predicted outcome of AIE-CSA-AKI. Each point represents a SHAP value for a feature and a specific observation, showing the distribution of the impacts each feature has on the model output. The color intensity indicates the feature value (red-high, blue-low).

**Figure 6:**
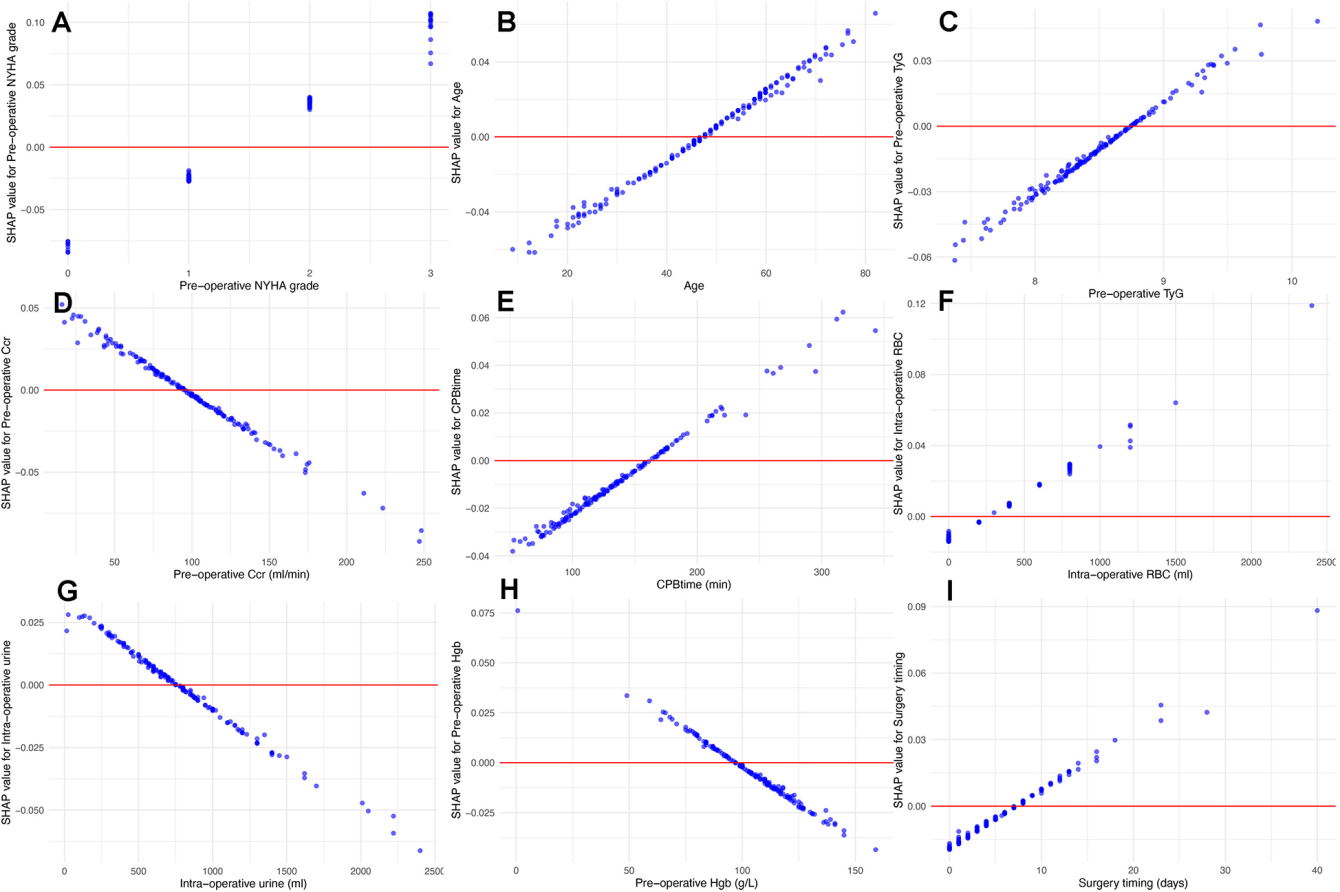
Univariate SHAP dependence plots for numeric and ordinal categorical features. These plots illustrate the relationship between feature values and their SHAP values. These plots reveal how specific feature values influence the model's predictions, particularly identifying cut-off points where the impact of a feature shifts from “protective” to “risk”. According to the plots, NYHA class III or IV **(A)**, age > 46 **(B)**, TyG index > 8.75 **(C)**, Ccr < 90 ml/min **(D)**, CPB time > 160 min **(E)**, intra-operative red blood cell transfusion > 300 ml **(F)**, intra-operative urine output < 750 ml **(G)**, pre-operative albumin < 97 g/L **(H)**, and diagnosisto-surgery gap over 7 days **(I)** were associated with high AKI risk.

We calculated SHAP values for all features in the validation set. Based on the mean SHAP values, we plotted a bar chart to represent the top 15 important features (Figure 4). A SHAP summary plot was also created to illustrate how specific features influence the predicted outcome (Figure 5). Additionally, univariate SHAP dependence plots were utilized to demonstrate the cut-off value for each numeric or ordinal categorical feature (Figure 6). The top 15 important features associated with AIE-CSA-AKI include valve replacement, pre-operative hypertension, large vegetations, NYHA class III or IV, alcoholism, age > 46, post-operative LCOS, TyG index > 8.75, Ccr < 90 ml/min, CPB time > 160 min, intra-operative red blood cell transfusion > 300 ml, intra-operative urine output < 750 ml, pre-operative hemoglobin < 97 g/L, a diagnosis-to-surgery gap of over a week, and male sex.

## Discussion

In this study, we trained and compared various machine learning algorithms to predict AIE-CSA-AKI, using data from 527 patients with active infective endocarditis who underwent cardiac surgery at our institution. Among the six algorithms tested, the RBF-SVM model demonstrated superior discrimination, achieving the highest AUC of 0.771 (CI, 0.698–0.844), surpassing both the Cleveland Clinic Score and the Mehta Score (10). Analysis of the top 15 important features, based on mean SHAP values, revealed that 10 are pre-operative, four are intra-operative, and only one, LCOS, is post-operative. This distribution suggests that, relying on this machine learning model, it is possible to predict AIE-CSA-AKI pre-operatively with reasonable accuracy. To our knowledge, this is the first study to compare multiple machine learning algorithms and develop a predictive model specifically tailored to AIE-CSA-AKI.

The advantage of our study lies in our institution's extensive database of patients with active infective endocarditis, which is sufficiently large to train machine learning models incorporating a significant number of features. Initially, 39 features were selected, primarily based on previous studies focusing on CSA-AKI. Additionally, we included features specifically associated with active infective endocarditis, such as large vegetations, embolisms, and infective shock, along with features indicative of the systemic inflammatory level, such as CRP, SIRI, SII, etc. Most of these features are pre-operative to avoid reverse causality, thereby enhancing the interpretability and clinical applicability of the model. The objective of feature selection was to ensure that the number of included features did not exceed the limit imposed by the sample size. In this process, the use of LASSO regression helped to prevent the dilution of features with internal correlations (11).

RBF-SVM stood out among the six tested algorithms. By employing the Radial Basis Function kernel, this algorithm transforms the input space into a higher-dimensional space, facilitating linear separation and proving especially effective for non-linear data (12). However, its characteristic of non-linear mapping, unlike logistic and tree models, renders the RBF-SVM model more akin to a “black box” that is difficult to interpret directly. To address this, we calculated SHAP values for all features in the validation set to explain how feature values impact the model's predicted result. The feature value where SHAP equals zero was used to represent the cut-off value for numeric and ordinal categorical features.

Among the top 15 important features, “valve replacement” was identified as the most significant. As defined in the “Methods” section, “valve replacement” refers to the replacement of one or more valves during surgery. In cases of AIE, we perform valve replacement when the infected area has expanded, the defect after debridement is large, and one or more valves are unrepairable. This classification includes all complex surgical approaches, such as double or triple valve replacement, aortic root replacement, and commando operations. In conclusion, “valve replacement” in this context indicates a more complex surgical approach. Moreover, valve replacement often indicates a more severe infective lesion, typically associated with a more serious systemic infection. Thus, the feature “valve replacement” not only reflects the complexity of the surgery but also the severity of the systemic infection, justifying its status as the most important feature.

Unlike CSA-AKI, the incidence of AIE-CSA-AKI is additionally influenced by systemic infection and inflammatory responses. This influence is evident in several key features of the model, including large vegetations, pre-operative CRP, SII, and SIRI. Notably, SII and SIRI are two recently identified biomarkers derived from blood cell counts. Research has shown that both SII and SIRI are effective in predicting adverse outcomes in patients with cardiovascular diseases (13–15), as well as in forecasting cardiovascular events and all-cause mortality in the general population (16, 17).

Surgical timing was identified among the top 15 important features of the model. The SHAP dependency plot showed that the timing of surgery, when performed within 7 days of diagnosis, altered the model's prediction direction. Early surgery for AIE patients turned out to be related to a lower prospective risk of CSA-AKI. Previous CSA-AKI models, including the Cleveland Clinic Score and the Simplified Renal Index (18), identified “non-selective” and “emergent” surgeries as risk factors for CSA-AKI. Our study appears to conclude the opposite. This contradiction is directly related to the significant difference between active infective endocarditis and non-infective structural cardiac diseases. In cases of AIE, as an active infection, the earlier the infected tissue is surgically removed, the lower the risk of post-operative mortality or morbidity. This supports the recommendation that AIE should be treated surgically as soon as possible.

Other important features in the model include NYHA class III or IV, pre-operative hypertension, age over 46, post-operative LCOS, pre-operative Ccr less than 90 ml/min, CPB time exceeding 160 min, intra-operative red blood cell transfusion greater than 300 ml, intra-operative urine output less than 750 ml, and pre-operative hemoglobin below 97 g/L. These findings are largely consistent with previously developed models for CSA-AKI (3–6).

## Study limitations

This study has several limitations. Firstly, being a single-center, retrospective study with a relatively small sample size, the performance of our model may not be optimal when applied to patient populations from different institutions or with varying characteristics. Hence, external validation is necessary to ensure its generalizability and to prevent overfitting. Secondly, the initial database features were selected manually, which may have led to the omission of some potentially important features. Thirdly, the limited number of positive samples constrained the number of features that could be included in the model. Consequently, the feature selection process might have overlooked some significant features. In conclusion, patients with AIE represent a unique subgroup among all cardiac surgery patients. Future studies are required to further explore the applicability of machine learning models to this specific group.

## Conclusion

Compared to CSA-AKI, AIE-CSA-AKI presents a more complex etiology, encompassing both the severity of local infection and the level of systemic inflammation. Machine learning models have shown efficacy in predicting AIE-CSA-AKI, suggesting that their use could enhance risk stratification and peri-operative management.

## Data Availability

The original contributions presented in the study are included in the article/Supplementary Material, further inquiries can be directed to the corresponding author.
